# Rural-Urban Determinants of Receiving Skilled Birth Attendants among Women in Bangladesh: Evidence from National Survey 2017-18

**DOI:** 10.1155/2022/5426875

**Published:** 2022-12-08

**Authors:** Sohani Afroja, Abu Saleh Muhammad Nasim, Md. Salauddin Khan, Mohammad Alamgir Kabir

**Affiliations:** ^1^Department of Statistics, Jahangirnagar University, Savar, Dhaka 1342, Bangladesh; ^2^Statistics Discipline, Science Engineering and Technology School, Khulna University, Khulna 9208, Bangladesh

## Abstract

**Background:**

SBAs (skilled birth attendants) play a crucial role in reducing maternal mortality. The proportion of maternal healthcare in Bangladesh that receives quality care at birth has increased; the reasons for this are unknown. The purpose of this study is to see if there has been a change in the use of specific maternal healthcare indicators in urban and rural areas, as well as significant risk factors.

**Materials and Methods:**

The data set was extracted from a nationally representative survey based on a cross-sectional study, the Bangladesh Health and Demographic Survey (BDHS) 2017-18. The frequency distribution reveals the general state of SBAs. To identify the association, we performed the chi-square test. Finally, multiple logistic regression was used to analyse the factors associated with SBAs and determine the degree of SBAs disparity between urban and rural areas.

**Results:**

In Bangladesh, 53% of women received SBAs during childbirth, with urban and rural areas receiving 68.1 and 52.2 percent, respectively. Women with secondary (AOR: 1.79, CI: 1.05–3.08) and higher (AOR: 4.18, CI: 2.09–8.50) education were more likely to receive SBAs than women in urban areas who were illiterate. Husband's education, women's working status, wealth index, children's birth order, and number of ANC visit are significant factors in receiving SBSs in both urban and rural areas. Higher educated husbands are 1.83 times (AOR = 1.83, CI: 1.04–3.25, *p* = 0.037) and 1.82 times (AOR = 1.82, CI: 1.29–2.59, *p* = 0.001) more likely to attend skilled births than uneducated husbands in both urban and rural areas. Respondents from the richest families are more likely to attend skilled births than those from the poorest families in both urban and rural areas.

**Conclusion:**

During delivery, significant risk factors are substantially related to SBAs. More attention must be given to rural and illiterate populations, who are less likely to obtain these services, to minimize maternal and neonatal mortality. Special programs could be developed to raise awareness and facilitate the poor in receiving the basic necessities of maternal care.

## 1. Introduction

Maternal and child mortality is a major public health concern all over the world. The majority of complications during pregnancy and childbirth are unanticipated and occur during delivery and postpartum. This is why all pregnant women should have access to skilled birth attendants (SBAs), who will ensure that a normal birth goes smoothly and that any complications are discovered and reported as soon as possible to the appropriate healthcare institutions [1]. The “the single most critical factor in avoiding maternal fatalities” is SBAs' attendance at birth [2]. SBAs' presence at delivery is also critical in reducing stillbirths and boosting neonatal survival [1, 3].

Even though maternal mortality during pregnancy and delivery has decreased from 451,000 in 2000 to 295,000 in 2017, the current figure is still concerning [4, 5]. Every year, an estimated 6000 women die in Bangladesh as a result of inadequate maternal health care services due to a lack of resources [6]. Nevertheless, between 1990 and 2015, the maternal mortality rate (MMR) in Bangladesh fell by 69 percent, from 574 to 176 deaths per 100,000 live births [7]. Making matters worse, more than 2.6 million children died in their first month of life worldwide in 2016, with South Asia accounting for 39 percent of those deaths [8–11]. Every year, in worldwide, roughly 40% of all under-five-year-old children die within their first month of life [8, 12–15]. A skilled birth attendant is an essential component in reducing maternal and infant mortality [16, 17]. In September 2015, members of the United Nations (UN) approved 17 SDGs (Sustainable Development Goals), one of which is SDG-03 (3.1-3.2); it aims to reduce MMR to less than 70 per 100,000 people by 2030 and new-born mortality to 12 per 1,000 live births worldwide [18, 19]. Skilled birth attendants are health professionals such as doctors, nurses, and midwives who are trained to provide medical coverage to women and new-born's before and during birth to handle normal deliveries and diagnose, treat, or guide obstetric complications [20–22]. In Bangladesh, one out of every five births uses an SBA, although the percentage is much lower in slums and tribal areas [23]. The fourth Health Population and Nutrition Sector Development Program of the Government of Bangladesh aimed for 65 percent SBA usage during delivery by 2022 [24].

It is critical to identify the elements that influence SBA delivery. Previous research has found that variables at different levels, such as individual, family, or socioeconomic circumstances, influence SBAs' decision to deliver [7, 25–29]. Inequality exists in Bangladesh, both in rural and urban areas, as well as by area and division. The rural context of Bangladesh has been the primary focus of research on equity in the use of skilled birth attendants. The structure of health service delivery varies greatly between urban and rural areas in Bangladesh [30]. A study of a home-based SBA program in rural Bangladesh found that financial constraints are significant barriers to healthcare utilization. Only 16% of the poorest households used the program, compared with 63% of the richest quintiles [31]. The goal of this research was to see if there were differences in patterns between urban and rural areas in order to identify underprivileged areas and significant factors while also raising awareness.

## 2. Materials and Methods

### 2.1. Data Sources & Study Design

This study uses data from the most recent Bangladesh Demographic and Health Surveys (BDHS) from 2017-2018. It is a cross-sectional data set for reproductive age (15–49) women measured nationally [32]. Because this research relies on a secondary data source, ethical clearance is not required. The information was gathered after submitting an application to the distributing authority [33]. A two-stage stratified sampling method was used in the BDHS 2017-18 [33]. There were 675 randomly selected clusters, 250 of which were in urban areas and 425 in rural areas. In the second stage, 30 randomly selected households from each cluster were chosen. The survey consisted a total of 20127 completed interviews of ever-married women aged 15–49 years, with 7374 (36.64%) from urban areas and 12753 (63.36%) from rural areas. The adjusting dataset for this analysis included 4974 respondents chosen for the final research. 1333 (26.8%) of respondents were urban, while 3641 (73.2%) were rural.

### 2.2. Dependent and Predictor Variables

The study's dependent variable is the presence or absence of a skilled birth attendant (SBA) during delivery. For this purpose, skilled birth is defined as the presence of a skilled doctor, nurse, midwife, paramedic, or family welfare visitor during the delivery. The SBA's service variable is not directly held on to in the BDHS 2017-18 data [32]. This variable is created by reversely categorizing the utilization of skilled birth attendance information, with the value 0 for an unskilled birth attendant and 1 for a skilled birth attendant.

Based on previous research, ten independent variables were chosen for this study. The independent variables were classified and are shown in Table 1.

### 2.3. Statistical Analysis

First, we performed a univariate analysis to examine the frequency distribution for this study. In the case of bivariate analysis, the changes in associated variables are shown in relation to the cross-tabulation outcome variable. Because all variables in this study were categorical, bivariate analysis is used to perform a chi-square test [34]. The data were presented as a contingency table with one of the variables as rows and the other as columns in Table 2. The test statistic is a chi-square(*χ*^2^) random variable defined by the following equation:

(1) $$\begin{array}{c} \chi^{2}=\sum \frac{{\left(O_{rc}-E_{rc}\right)}^{2}}{E_{rc}}∼\chi_{df}^{2}\text{.} \end{array}$$

Multiple logistic regression was used to model determinants that significantly explain the SBAs in various aspects for multivariate analysis by equation (2) in Table 3. The logistic regression model can also be written in the form of a log of odd-

(2) $$\begin{array}{c} \ln\;\left(\frac{\hat{p}}{1-\hat{p}}\right)=X\beta \text{,} \end{array}$$

where *X* = (*X*_1_, *X*_2_ … *X*_*n*_), *β* = (*β*_0_, *β*_1_ … *β*_*n*_). $\hat{p}$ is the expected probability of occurrence of the outcome, *X* is the independent variable, and *β* is the regression coefficient. Equation (2) can be defined as

(3) $$\begin{array}{c} \ln\left[\frac{P\left(Y=m\right)}{P\left(Y=0\right)}\right]=\beta_{0}+\overset{n}{\underset{p=1}{\sum }}\beta_{p}X_{p}\text{,} \end{array}$$

where *m* = 1,2,…, *n* To find the log-odd ratio, the probability of each event is calculated. The odds ratio measures the incidence when the independent variable increases by one unit. The odds ratio is defined as

(4) $$\begin{array}{c} \frac{P\left(Y=m\right)}{P\left(Y=0\right)}=\exp\;\left(\beta_{0}+\overset{n}{\underset{p=1}{\sum }}\beta_{p}X_{p}\right)\text{.} \end{array}$$

The classification is to predict the women in the presence of skilled birth attendants. From the calculated coefficients, the probability of each sample is calculated. The probability is defined as *P*(*Y*=*m*)=exp (*g*(*x*))/1+∑exp (*g*(*x*)) while for the reference category, *P*(*Y*=0)=1/1+∑exp (*g*(*x*)).

## 3. Results and Discussion

### 3.1. Univariate Analysis

Table 4 lists the baseline characteristics of urban and rural respondents. The Dhaka division has 46.9% of the urban respondents. The majority of women (24.3%) have a higher education and are unemployed (71.6%). 45.6% of the respondents come from the richest family. More than half of the respondents (51.2%) women gave birth to their first child at the age of 19 or older, with the majority (59.0%) reporting ANC visits more than four times. 80.5% of respondents have had media exposure. One-third (34.4%) of the participants' husbands have secondary education in an urban area.

For the rural population, most of the respondents are from the Chittagong division (22.2%). Only several respondents have higher education (14.6%) and are unemployed (59.7%). 10.1% of the respondents belong to the richest family. More than half of the respondents (59.9%) women gave birth to their first child at 18 or below years, and most of them (48.2%) reported ANC visits more than 1–3. Majority of the respondents (60.4%) have media exposure. 35.3% participant's husbands had primary education in rural area.

**Figure 1:**
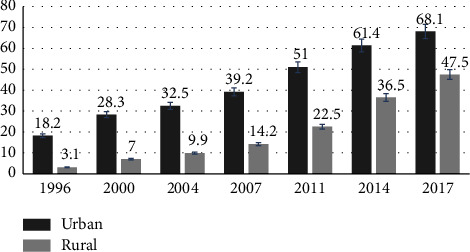
Percentage of received SBAs among urban and rural from 1996–2017.

In 1996-1997, the proportion of skilled birth attendants in urban and rural areas was 18.2 percent and 3.1 percent, respectively, according to Figure 1. It rises by 68.1 percent in urban areas and 47.5 percent in rural areas in Bangladesh, according to a 2017-18 survey. Thus, between 1996 and 2017, the number of skilled birth attendants increased gradually in both urban and rural areas. However, rural areas require additional attention.

### 3.2. Bivariate Analysis

Table 2 shows the bivariate correlation with individual characteristics for selected SBA predictors for urban and rural women. In urban areas, the majority of respondents who had experienced skilled birth are from Dhaka (73.3%) and Khulna (74.4%) divisions. Women who had skilled birth had higher education (93.5%) and were unemployed (72.3%); on the other hand, the majority of their husbands had higher education (92.3%). Women from the wealthiest families (86.7%) had more skilled births.

**Figure 2:**
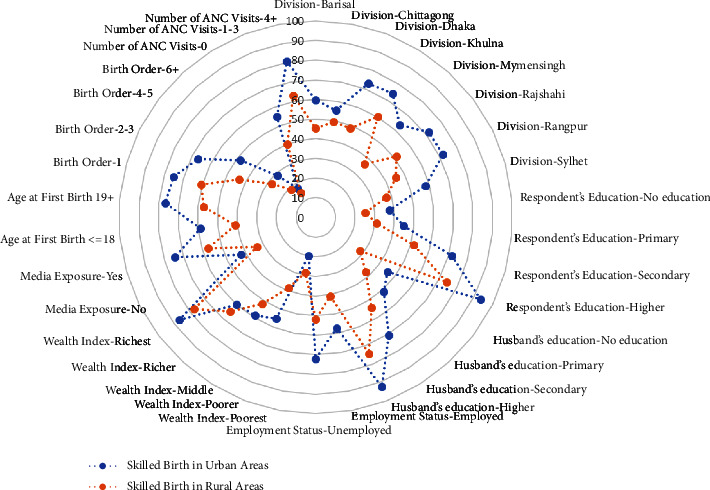
Prevalence of skilled birth attended in terms of urban and rural area in Bangladesh (radar plot).

The majority of respondents (77.0%) went to skilled birth and had their first child at the age of 19 or older (75.3%). Women who visited ANC more than four times (80.6%) and had media exposure (74.3%) had skilled births.  Figure 2 also showed that the percentage of skilled births was higher in urban areas than in rural areas, where the following factors were highly associated, such as education level for both husband and wife and wealth index.

For the rural areas, the Khulna division took the highest proportion of respondents who had a skilled birth (60.2%). Women who had a skilled birth had a higher education (74.3%) and were unemployed (52.0%); on the other hand, the majority of their husbands had a higher education (74.4%). Women from the wealthiest families (77.4%) had more skilled births. The majority of respondents (57.1%) attended a skilled birth and had their first child at the age of 19 or older (60.5%). Women who visited ANC more than four times (63.2%) and had media exposure (56.7%) had skilled births.

### 3.3. Multivariate Analysis

Table 3 displays the findings of a multiple logistic regression analysis conducted between skilled birth attendance and other independent variables in urban and rural areas. For the urban population, respondents with secondary education are almost 1.79 times (AOR = 1.79, 95% CI: 1.05–3.08, *p* = 0.035) and respondents with higher education are 4.17 times (AOR = 4.17, 95% CI: 2.08–8.48, *p* < 0.001) more likely to attend skilled birth compared to uneducated respondents. Women are almost 1.83 times (AOR = 1.83, 95% CI: 1.04–3.25, *p* = 0.037) more likely to attend a skilled births whose husbands are more educated than those whose husbands are uneducated. Employed women are 25% (AOR = .75, 95% CI: 0.57–0.99, *p* = 0.041), less likely to attend skilled births compared to unemployed women. Respondents from poorer families are almost 2.56 times (AOR = 2.56, 95% CI: 1.50–4.42, *p* < 0.001), the middle-income family almost 2.72 times (AOR = 2.72, 95% CI: 1.66–4.50, *p* < 0.001), the richer family 3.26 times (AOR = 3.26, 95% CI: 2.03–5.30, *p* < 0.001), and richest family almost 6.61 times (AOR = 6.61, 95% CI: 3.89–11.38, *p* < 0.001) more likely to attend skilled birth compared to poorest families. Respondents with 2-3 birth orders are almost 26% (AOR = 0.74, 95% CI: 0.56–0.97, *p* = 0.029) less likely to attend skilled birth compared to respondents with 1^st^ birth order. Respondents who have 1–3 ANC visits are almost 3.45 times (AOR = 3.45, 95% CI: 1.83–6.99, *p* < 0.001) and more than 4 visits are almost 7.45 times (AOR = 7.45, 95% CI: 3.91–15.19, *p* < 0.001) more likely to attend skilled birth compared to 0 visits.

For rural population, respondents from Mymensingh division are almost 41% (AOR = 0.59, 95% CI: 0.43–0.81, *p* = 0.001) and from Sylhet division are 36% (AOR = 0.64, 95% CI: 0.46–0.88, *p* = 0.006) less likely to attend skilled birth than respondents from Barisal division.

Women are almost 1.82 times (AOR = 1.82, 95% CI: 1.29–2.59, *p* = 0.001) more likely to attend a skilled birth where husbands are more educated than those whose husbands are uneducated. Employed women are 22% (AOR = 0.678, 95% CI: 0.66–0.92, *p* = 0.004), less likely to attend skilled births compared to unemployed women. Respondents from a middle-income family are almost 1.56 times (AOR = 1.56, 95% CI: 1.21–2.00, *p* = 0.001), the richer family almost 2.02 times (AOR = 2.02, 95% CI: 1.53–2.68, *p* < 0.001), and richest family almost 3.07 times (AOR = 3.07, 95% CI: 2.14–4.44, *p* < 0.001) more likely to attend skilled birth compared to poorest families. Respondents who have media exposure are 1.32 times (AOR = 1.32, 95% CI: 1.11–1.57, *p* = 0.002) more likely to attend skilled birth than respondents with no media exposure. Respondents over 19 are almost 1.29 times (AOR = 1.29, 95% CI: 1.10–1.53, *p* = 0.002) more likely to attend skilled birth than respondents aged less than 18 years. Respondents with 2-3 birth orders are almost 27% (AOR = 0.63, 95% CI: 0.53–0.75, *p* < 0.001), 4-5 birth orders are 51% (AOR = 0.49, 95% CI: 0.36–0.66, *p* < 0.001), and 6+ birth orders are almost 56% (AOR = 0.44, 95% CI: 0.22–0.81, *p* < 0.011) less likely to attend skilled birth compared to respondents more than 1^st^ birth orders. Respondents who have 1–3 ANC visits are almost 2.55 times (AOR = 2.55, 95% CI: 1.81–3.69, *p* < 0.001) and more than 4 visits are almost 5.14 times (AOR = 5.14, 95% CI: 3.61–7.49, *p* < 0.001) more likely to attend skilled birth compared to 0 visits.

## 4. Discussion

This study identified various socio-demographic and economic features, indicating that incorporating individual-level aspects may not be sufficient for evaluating health care services. This study aims to understand the impact of influencing cases on skilled help during delivery care in urban and rural areas of Bangladesh using data from a national demographic and health survey conducted in 2017-18. Our findings indicate that skilled birth attendance is substantially correlated with place of residence. Where skilled, birth attendance is less likely among rural residents. A similar result was found in a study conducted in India, Bangladesh, and Nepal [16]. Different studies in Bangladesh have also shown similar results [35, 36]. The division has a significant association with skilled birth attendance. Another study in Afghanistan and Kazakhstan coincides with the present study [37–39]. Low usage of SBAs services in some areas in Bangladesh is attributable to low-quality services, service unavailability and inaccessibility, lack of support workers, pharmaceutical shortages, equipment, and lack of support awareness.

The respondent's level of education is a significant determinant of skilled birth attendance and respondents with greater education are more likely to engage in skilled birth during delivery in both rural and urban. A study conducted in Bangladesh, India, and Nepal came to the same conclusion [16]. A study in Bangladesh does not coincide with this study [40]. Nevertheless, some other research showed a similar result [1, 35, 36, 41]. Women who have received an education are often well-informed about numerous health challenges, including difficulties in finding conventional, nonscientific, and inexpensive remedies. It makes them reconsider using the same service that their predecessors did.

The wealth index has a significant association with skilled birth attendance. Women with higher economic positions in both urban and rural were more likely to hire safe delivery services when compared to those with lower economic status. Research in Nepal, India, and Afghanistan showed the same result [2, 4]. Previous studies in Bangladesh have also stated similar findings [35, 36, 40, 42]. The cost of SBA services is inaccessible to households in the lowest wealth quintile. They avoid going to hospital or using other types of healthcare since doing so would require them to forgo purchasing necessities like food and clothing. Because few skilled professionals work in Bangladesh's remote regions, SBAs prefer to assist those houses that pay them well.

Respondent's age at 1^st^ birth is associated with skilled birth attendance in both urban and rural areas. Other researchers have come to the same conclusion [16, 26, 36, 43]. Many young women who had their first child while they were teenagers may have trouble getting SBA. These barriers include the price of maternal healthcare services, stigma, and a poor perception of healthcare workers.

Birth order is a prominent factor where this research shows that women with the first birth order were more anxious about using SBAs than women with other birth orders, according to the observed birth order predictors. The similarity was found in several research studies conducted in different countries [16, 36, 44, 45]. Women with 1^st^ or 2^nd^ birth order appear to be more conscious about their children and tend to attend skilled births in urban and rural. Nevertheless, as the birth order increases, their consciousness becomes lower.

According to our findings, the expectations of using SBAs were higher in women who had more antenatal care (ANC) visits. The increased ANC usage and SBAs among urban and rural during the recent delivery had a strong relationship, consistent with a prior study [40]. This might mean that ANC examinations can help respondents understand the need for experienced caregivers to deliver their babies. Previous research has revealed that professional maternity services pay more attention to ANC visits [46, 47]. There is a higher concentration of ANC visits in these populations, indicating that they are more aware of the benefits of employing SBAs than traditional birth attendants.

Individual predictors of media access and women using high-density mass media in both urban and rural access strongly correlated with SBAs' care. A previous study established a link between media exposure and SBAs' care similarly [36, 40]. One likely explanation is that after watching television, reading magazines or newspapers, or listening to the radio, people are easily encouraged to employ delivery assistance. As a result, pregnant women should be encouraged to use mass media such as television, radio, magazines, and newspapers to reach out to support of safe and professional delivery techniques.

This study also found a considerable influence of the respondent profession on competent health care during delivery among urban and rural women. In both urban and rural areas, our study indicated that working women are less likely to hire skilled delivery attendants.

In this study, SBAs discovered a link between the husband's higher education and encouragement for delivery. Previous research in Bangladesh and elsewhere has found a high level of agreement between education, professional delivery support, and other maternal health treatments [35, 40]. Advances in spouses' educational levels prompted the likelihood of SBAs' care. This may increase decision-making involvement by increasing economic independence and autonomy, intensifying health-seeking behaviour and expanding social capital via widening social interfaces. A well-educated spouse may have an educated impression of his neighbours, exposing them to those who want to be more involved in choosing the SBAs' care.

## 5. Conclusions

This study discovered several characteristics associated with SBAs during delivery in Bangladesh. According to our findings, certain characteristics such as age, education, husband's and respondent's education, wealth index, employment, and ANC visits have a significant influence on SBAs. SBAs during pregnancy are associated with characteristics such as residency, media access, more than four ANC visits, and respondents from well-educated and wealthy families. The findings of the study are critical for informing the government, health planners, and other public health stakeholders in Bangladesh about how to reduce socioeconomic disparities in SBA services among underprivileged and uneducated women. Rural women may require education, knowledge, media access, and monetary assistance to increase their use of SBA services. It is also a good idea to plan awareness events around SBA-related variables. This could help to reduce maternal and infant mortality in Bangladesh.

## Figures and Tables

**Table 1:** Variable definitions and coding for analysis.

Variable name	Code	Variable category
Skilled birth attendants	0	Unskilled
	1	Skilled

Place of residence	1	Urban
	2	Rural

Division	1	Barisal
	2	Chittagong
	3	Dhaka
	4	Khulna
	5	Mymensingh
	6	Rajshahi
	7	Rangpur
	8	Sylhet

Respondent's education level	0	No education
	1	Primary
	2	Secondary
	3	Higher

Husband's education level	0	No education
	1	Primary
	2	Secondary
	3	Higher

Respondent's employment status	0	Unemployed
	1	Employed

Wealth index	1	Poorest
	2	Poorer
	3	Middle
	4	Richer
	5	Richest

Media exposure	1	No
	2	Yes

Respondent's age at first birth	1	<20 age
	2	20–34
	3	35–49

Children's birth order	1	1
	2	2-3
	3	4-5
	4	6+

Number of ANC visits	1	0
	2	1–3
	3	4+

**Table 2:** Prevalence of skilled birth attended in terms of urban and rural area in Bangladesh for the selected predictors of women.

Variable	Urban		*p* value^1^	Rural		*p* value^1^
	Unskilled	Skilled		Unskilled	Skilled	
Division						
Barisal	19 (40.4)	28 (59.6)	<0.001	128 (54.5)	107 (45.3)	<0.001
Chittagong	110 (44.5)	137 (55.5)		410 (50.4)	400 (49.6)	
Dhaka	167 (26.7)	458 (73.3)		333 (51.2)	317 (48.8)	
Khulna	27 (26.0)	77 (74.0)		139 (39.8)	210 (60.2)	
Mymensingh	27 (36.5)	47 (63.5)		222 (63.4)	128 (36.6)	
Rajshahi	29 (28.2)	74 (71.8)		231 (48.6)	244 (51.4)	
Rangpur	21 (28.0)	54 (72.0)		248 (54.7)	205 (45.3)	
Sylhet	24 (42.1)	33 (57.9)		202 (62.9)	119 (37.1)	
Respondent's education						
No education	53 (62.4)	32 (37.6)	<0.001	168 (75.0)	56 (25.0)	<0.001
Primary	185 (55.2)	150 (44.8)		714 (69.0)	321 (31.0)	
Secondary	166 (28.2)	422 (71.8)		892 (48.2)	957 (51.8)	
Higher	21 (6.5)	304 (93.5)		137 (25.7)	397 (74.3)	
Husband education level						
No education	71 (54.2)	60 (45.8)	<0.001	394 (71.8)	155 (28.2)	<0.001
Primary	192 (48.6)	203 (51.4)		802 (62.5)	481 (37.5)	
Secondary	135 (29.4)	324 (70.6)		568 (46.0)	668 (54.0)	
Higher	27 (7.7)	322 (92.3)		146 (25.6)	425 (74.4)	
Respondent's employment status						
Employed	161 (42.5)	218 (57.5)	<0.001	868 (59.2)	599 (40.8)	<0.001
Unemployed	264 (27.7)	690 (72.3)		1043 (48.0)	1132 (52.0)	
Wealth index						
Poorest	93 (80.2)	23 (19.8)	<0.001	650 (71.4)	261 (28.6)	<0.001
Poorer	38 (44.7)	47 (55.3)		571 (61.5)	358 (38.5)	
Middle	65 (41.4)	92 (58.6)		380 (48.0)	411 (52.0)	
Richer	147 (40.1)	220 (59.9)		227 (35.2)	417 (64.8)	
Richest	81 (13.3)	527 (86.7)		83 (22.6)	284 (77.4)	
Media exposure						
No	150 (57.5)	111 (42.5)	<0.001	959 (66.5)	483 (33.5)	<0.001
Yes	275 (25.7)	797 (74.3)		952 (43.3)	1247 (56.7)	
Respondent's age at first birth						
≤18	268 (41.2)	382 (58.8)	<0.001	1284 (58.8)	898 (41.2)	<0.001
19+	157 (23.0)	526 (77.0)		626 (42.9)	832 (57.1)	
Birth order						
1	134 (24.7)	408 (75.3)	<0.001	531 (39.5)	815 (60.5)	<0.001
2-3	223 (33.3)	446 (66.7)		1021 (56.5)	786 (43.5)	
4-5	53 (52.0)	49 (48.0)		294 (71.9)	115 (28.1)	
6+	15 (74.1)	6 (28.6)		65 (81.3)	15 (18.8)	
Number of ANC visits						
0	57 (82.6)	12 (17.4)	<0.001	280 (85.6)	47 (14.4)	<0.001
1–3	215 (45.1)	262 (54.9)		1057 (60.2)	698 (39.8)	
4+	153 (19.4)	634 (80.6)		573 (36.8)	986 (63.2)	

**Table 3:** Multiple logistic regression analysis of associated factors of skilled birth attended in terms of rural and urban women in Bangladesh.

Factors	SBA (urban)				SBA (rural)			
	AOR	95% CI		*p* value^1^	AOR	95% CI		*p* value^1^
		Lower	Upper			Upper	Lower	
Division								
Barisal (ref)	1				1			
Chittagong	0.81	0.49	1.33	0.406	0.77	0.57	1.05	0.105
Dhaka	1.23	0.76	1.99	0.398	0.72	0.51	1.02	0.064
Khulna	1.41	0.82	2.44	0.216	1.18	0.84	1.66	0.343
Mymensingh	1.26	0.73	2.19	0.406	0.59	0.43	0.81	**<0.001**
Rajshahi	1.38	0.78	2.44	0.269	0.90	0.64	1.25	0.519
Rangpur	1.37	0.78	2.44	0.277	0.79	0.57	1.10	0.158
Sylhet	0.94	0.55	1.59	0.805	0.64	0.46	0.88	**<0.006**
Respondent's education level								
No education (ref)	1				1			
Primary	0.99	0.58	1.69	0.971	0.94	0.64	1.40	0.768
Secondary	1.79	1.05	3.08	**<0.035**	1.24	0.84	1.84	0.284
Higher	4.18	2.09	8.50	**<0.001**	1.55	0.98	2.48	0.064
Husband's education level								
No education (ref)	1				1			
Primary	0.97	0.64	1.47	0.883	1.12	0.87	1.44	0.389
Secondary	1.21	0.78	1.86	0.393	1.17	0.89	1.54	0.262
Higher	1.83	1.04	3.25	**<0.037**	1.82	1.29	2.59	**<0.001**
Respondent's employment status								
Unemployed (ref)	1				1			
Employed	0.75	0.57	0.99	**<0.041**	0.78	0.66	0.92	**<0.004**
Wealth index								
Poorest (ref)	1				1			
Poorer	2.56	1.50	4.42	**<0.001**	1.15	0.92	1.43	0.227
Middle	2.72	1.66	4.50	**<0.001**	1.56	1.21	2.00	**<0.001**
Richer	3.26	2.03	5.30	**<0.001**	2.02	1.53	2.68	**<0.001**
Richest	6.61	3.89	11.38	**<0.001**	3.07	2.14	4.44	**<0.001**
Media exposure								
No (ref)	1				1			
Yes	1.17	0.85	1.62	0.338	1.32	1.11	1.57	**<0.002**
Respondent's age at 1^st^ birth								
≤18 (ref)	1				1			
19+	1.23	0.95	1.59	0.124	1.29	1.10	1.53	**<0.002**
Children's birth order								
1 (ref)	1				1			
2-3	0.74	0.56	0.97	**<0.028**	0.63	0.53	0.75	**<0.001**
4-5	0.87	0.55	1.38	0.548	0.49	0.36	0.66	**<0.001**
6+	0.70	0.24	1.87	0.487	0.44	0.22	0.81	**<0.011**
Number of ANC visit								
0 (ref)	1				1			
1–3	3.45	1.83	6.99	**<0.001**	2.55	1.81	3.69	**<0.001**
4+	7.45	3.91	15.19	**<0.001**	5.14	3.61	7.49	**<0.001**

**Table 4:** Frequency distribution in terms of urban and rural women for selected variables.

Factors	Urban		Rural	
	Frequency (*n*)	Percentage (%)	Frequency (*n*)	Percentage (%)
SBA				
Skilled	908	68.1	1911	47.5
Unskilled	425	31.9	1730	52.5
Division				
Barisal	47	3.5	235	6.5
Chittagong	248	18.6	807	22.2
Dhaka	625	46.9	650	17.8
Khulna	104	7.8	349	9.6
Mymensingh	74	5.5	350	9.6
Rajshahi	103	7.7	476	13.1
Rangpur	75	5.6	454	12.5
Sylhet	57	4.3	321	8.8
Respondent's education				
No education	86	6.4	224	6.1
Primary	335	25.1	1035	28.4
Secondary	588	44.1	1849	50.8
Higher	325	24.3	533	14.6
Husband education level				
No education	131	9.8	549	15.1
Primary	394	29.6	1284	35.3
Secondary	459	34.4	1237	34.0
Higher	349	26.2	571	15.7
Respondent's employment status				
Unemployed	954	71.6	2175	59.7
Employed	379	28.4	1466	40.3
Wealth index				
Poorest	116	8.7	911	25.0
Poorer	85	6.4	929	25.5
Middle	158	11.8	791	21.7
Richer	367	27.5	644	17.7
Richest	607	45.6	367	10.1
Media exposure				
No	261	19.5	1442	39.6
Yes	1073	80.5	2198	60.4
Respondent's age at first birth				
≤18	650	48.8	2182	59.9
19+	683	51.2	1459	40.1
Child's birth order				
1	542	40.6	1345	36.9
2-3	669	50.2	1807	49.6
4-5	101	7.6	409	11.2
6+	21	1.6	79	2.2
Number of ANC visits				
0	69	5.2	327	9.0
1–3	477	35.8	1755	48.2
4+	787	59.0	1559	42.8

## Data Availability

https://dhsprogram.com/methodology/survey/survey-display-536.cfm.
